# The Role of Traditional Obesity Parameters in Predicting Frailty among Coronary Artery Disease Patients Undergoing Cardiac Catheterization

**DOI:** 10.1155/2022/8676274

**Published:** 2022-09-12

**Authors:** Audai A. Hayajneh, Islam M. Alhusban, Mohammad Rababa

**Affiliations:** Adult Health-Nursing Department, Faculty of Nursing, Jordan University of Science and Technology, P.O. Box: 3030, Irbid, Jordan

## Abstract

**Background:**

Obesity has been reported to be associated with frailty and coronary artery disease (CAD).

**Objective:**

The present study aimed to investigate the role of the seven traditional obesity parameters body mass index (BMI), waist-height ratio (WHtR), waist-hip ratio (WHR), body adiposity index (BAI), body shape index (BSI), waist circumference (WC), and hip circumference (HC) in the prediction of frailty among CAD patients undergoing cardiac catheterization.

**Design:**

A secondary data analysis was conducted. *Setting*. Three main hospitals were located at the northern and middle regions of Jordan. *Participants.* 220 hospitalized patients undergoing cardiac catheterization were recruited. *Measurements*. The traditional obesity parameters were measured using an anthropometric tape and weight scale and frailty was measured using the Tilburg Frailty Indicator (TFI). Data were analyzed using bivariate Pearson's correlation and forward linear regression analysis.

**Results:**

Total cholesterol, HC, triglycerides, age, random blood sugar, and WC had significant positive associations with and were predictors of frailty (*p* < 0.05). The model of the seven predictors explained 32.4% of the variance in frailty (*p* = 0.02).

**Conclusion:**

The incidence of frailty can be predicted by the increase in total cholesterol, HC, triglycerides, age, random blood sugar, and WC. The results of this study may help healthcare providers, including nurses, to identify the factors that could lead to frailty among CAD patients undergoing cardiac catheterization.

## 1. Introduction

Obesity is conceptually described as the excessive accumulation of fatty tissue across the human body, predisposing patients to several health risks [1]. The World Health Organization (WHO) operationally defines obesity by body mass index (BMI), which is the result of dividing weight in kilograms by the square of height in meters, into obese stage I (BMI of 30–34.9), obese stage II (BMI of 35–39.9), and obese stage III (BMI of 40 or more) [1].

Obesity is considered a strong contributing factor for the progression of frailty [2]. Frailty is conceptually defined as the loss or decrease in a patient's normal physiological, physical, mental, or psychological functioning, affecting their quality of life, productivity, and overall medical condition [3].

Several factors have been reported to be associated with the incidence of frailty. As compared to younger people, older people have a higher tendency to become frail, which may be due to their increased risks of having chronic diseases like diabetes mellitus, hypertension, and dyslipidemia [4]. Further, Kojima and colleagues [5] found that smoking had a positive relationship with frailty, and another study reported a positive relationship between depression and frailty [6]. Contradictory findings have been reported regarding the relationship between gender and frailty. Zasadzka and colleagues [7] reported that males had higher susceptibility to be frail than did females, whilst Zhang and colleagues [8] found that females were more susceptible.

Thus, obesity was revealed as one of the major risk factors for coronary artery diseases (CADs) among patients undergoing cardiac catheterization. Frailty might be coexistent with frailty among those risky patients. Thus, the detection of frailty among patients with CADs undergoing cardiac catheterization should be based on holistic approach, using not merely one instrument, but the traditional obesity parameters as well as health and biomarkers might provide a better scope of frailty. Therefore, this study aimed to investigate the role of the seven traditional obesity parameters BMI, waist-height ratio (WHtR), waist-hip ratio (WHR), body adiposity index (BAI), body shape index (BSI), WC, and hip circumference (HC) as well as health and biomarkers in predicting frailty among CAD patients undergoing cardiac catheterization.

## 2. Methods

### 2.1. Research Design

A secondary data analysis was adopted from an original study [9]. The aim of this study was to determine the role of the traditional obesity parameters in predicting frailty among hospitalized CAD patients undergoing cardiac catheterization.

### 2.2. Setting and Sample

The study participants were recruited from two major referral hospitals in the middle and north regions of Jordan. Data were collected from the participating patients before they underwent cardiac catheterization. Data collection took place in the hospital wards, including medical wards, coronary care units, intensive care units, and emergency departments, before the patients were transferred to the catheterization laboratories. The required sample size was calculated as 172 patients using *G* power with the following settings: F test as family test and linear hierarchical multiple regression with effect size 0.15, alpha error probability of 0.05, power of 0.8, and number of predictors of 25. To account for any possible dropouts, an additional 25% of the required sample size was added, and a total of 220 participants were recruited using convenience sampling.

The inclusion criteria included being a Jordanian male or female aged 18 years or over and being a hospitalized patient undergoing either elective or urgent cardiac catheterization. Meanwhile, the exclusion criteria included being a patient with severe organ disease, such as severe liver disease and renal failure, being pregnant, having undergone coronary artery bypass graft surgery, having an autoimmune disease, being an immunosuppressed patient who has undergone organ transplant, and being a cancer patient.

### 2.3. Data Collection

The independent variables, which included the obesity parameters and the basic data (i.e., weight, height, WC, and HC), were recorded by well-trained registered nurses. These nurses had completed three training sessions of one hour each, delivered by the primary researcher. The data were collected at the admission assessment before cardiac catheterization using a flexible anthropometric tape for WC and HC, and a rigid anthropometric tape for height and weight. Participants were recruited during the period from March 18, 2021, to July 18, 2021.

### 2.4. Measurements

Data pertaining to the participants' sociodemographic and health characteristics were collected from the patients themselves or from their medical files. These characteristics included age, gender, education, marital status, income, employment, smoking status, blood pressure, and daily activity based on the number of daily steps. Serology tests were also collected, including low density lipoprotein (LDL), high density lipoprotein (HDL), triglyceride, high-sensitivity C-reactive protein (HS-CRP), random blood sugar (RBS), and hemoglobin A1c (HbA1c).

Anxiety and depression levels were analyzed as continuous variables (score from 1 to 21) and measured using a psychometric tool. The Hospitalized Anxiety and Depression Scale (HADS), developed by Zigmond and colleagues [10], is a tool for measuring anxiety and depression in hospitals. Good internal consistency has been reported for the HADS, with Cronbach's alpha values of 0.87 for anxiety and 0.81 for depression [11]. The Arabic version of the HADS, developed by Terkawi and colleagues [12], had Cronbach's alpha values of 0.83 for anxiety and 0.77 for depression [12]. This Arabic version was used in the present study to measure patients' anxiety and depression before undergoing cardiac catheterization. The total possible anxiety and depression scores are classified as follows: 0–7 = normal, 8–10 = borderline abnormal (borderline case), and 11–21 = abnormal case (has anxiety or depression).

As with regard to the traditional obesity parameter measurements, weight was measured to the nearest 0.1 kg using a rigid measurement device and with the participants wearing their hospital gowns. Waist circumference was measured at the midpoint between the lowest rib margin and the level of the anterior superior iliac crest, using a flexible anthropometric tape and with measurements rounded to the nearest 0.1 cm. Hip circumference measurements were taken at the greatest protrusion of the gluteal muscles and also rounded to the nearest 0.1 cm. Waist hip ratio was calculated as WC/HC, and WHtR as WC/height. The formula (WC (cm))/(〖BMI〗 ^ 2/3) × (〖Height (m)〗 ^ 0.5) was used to calculate BSI, and the formula (weight (kg))/〖height (m)〗 ^ 2) was used to calculate BMI. The formula (HC (cm))/〖height (*m*) 〗 ^ ^1.5^–18) was used to calculate BAI.

Frailty was measured using the Tilburg Frailty Indicator (TFI) prior to the patients undergoing cardiac catheterization. The TFI is a 15-item self-report questionnaire used to measure frailty [13]. The TFI has four main domains, and good correlation coefficient (*r*) values have been reported for the physical (−0.71), psychological (*r* = −0.68), social (*r* = −0.40), and environmental (*r* = 0.54) domains (*p* < 0.001 for all domains) [13]. Furthermore, a good test-retest reliability of 0.79 was reported for the TFI within a one-year period, with a value of 0.78 reported for the physical domain, 0.67 for the psychological domain, and 0.76 for the social domain [13].

An acceptable Cronbach's alpha value (0.771) [14] has been reported for the Arabic translated version of the TFI, which was used in the present study. Also, the Arabic version of the TFI has good reliability measured by determining the KR-20 values as follows: 0.744 (Physical-TFI), 0.46 (Psychological-TFI), 0.39 (Social-TFI), and 0.77 (Total-TFI) [14]. Patients are considered frail if they have a score ≥5, with the total possible score being 15 [15].

### 2.5. Statistical Analysis

Means and standard deviations were used to describe the continuous variables, such as age, and frequencies and percentages were used to describe the categorical variables, such as gender. Bivariate Pearson's correlation was employed to assess the correlation (strength of relationship between variables) between the obesity parameters, socio-demographic and health variables, and frailty. *T* test was used for variables with two categories, such as gender, and “ANOVA test” was used for variables with more than two categories, such as employment status, marital status, and educational level. Forward linear regression was employed to assess the ability of the obesity parameters and sociodemographic and health variables to predict frailty. The Statistical Package for the Social Sciences software version 25 (SPSS) was used to analyze the study data. A significance level of 0.05 was used in the current study.

### 2.6. Ethical Considerations

After obtaining institutional review board approval from Jordan University of Science and Technology and approval from the selected hospitals, consent forms were obtained from the participants prior to their participation. These forms outlined the participants' rights to withdraw from the study at any time without consequences, in addition to the intended benefits and risks of participation. The principal researcher and trained research assistants explained the study's purpose, procedures, intended benefits, and potential risks to all of the participants. The participants were provided with time to read the consent forms and ask any questions about the study prior to them deciding whether to participate in the study.

## 3. Results

The average age of the participants was 49.9 years (SD ± 11.7), with ages ranging between 24 and 90 years. Around two-thirds (73.2%) of the participants were male, and the majority of the participants (62.3%) were married. As for the employment status, approximately half of the participants (49.1%) were employed. Further, 38.2% of the participants were nonsmokers, while the rest (61.8%) were smokers. Around two-thirds (72.7%) of the participants had anxiety, and around half (45.5%) had depression (see Table 1).

Correlations (Pearson's correlation) between the traditional obesity parameters and frailty were built in order to assess the relationships between these parameters. Frailty was found to have significant positive weak correlations with weight, WC, HC, and BAI, as shown in Table 2.

To test the relationship between the model of obesity parameters, health variables, and sociodemographic variables and frailty, statistical analysis was carried out using a forward regression analysis as shown in Table 3. Model (1), which had one predictor (total cholesterol), explained 9% of the variance in frailty (*p* < 0.001), while model (7), which had seven predictors, explained 32.4% of the variance (*p*=0.02). Model (7) illustrated that increased total cholesterol by 7.1 mg/dl (*β* (0.288)*∗* SD (24.7)), increased HC by 8.3 cm (*β* (0.62)*∗* SD (13.4)), increased age by 8.3 years (*β* (0.245)*∗* SD (11.4)), increased triglycerides by 8.6 mg/dl (*β* (0.25)*∗* SD(34.3), increased RBS by 4.9 mg/dl (*β* (0.214)*∗* SD(23)), or increased WC by 4.4 cm (*β* (0.329)*∗* SD(13.3)) increased frailty by 1 score (*p* < 0.05). However, increased HDL by 0.7 mg/dl (*β* (0.146)*∗* SD (4.8)) decreases frailty by 1 score (*p*=0.02).

## 4. Discussion

The relationships between frailty and its possible predictors (i.e., obesity parameters and sociodemographic and health variables) were investigated. It was concluded that there were strong and moderate relationships between frailty and its possible predictors among CAD patients undergoing cardiac catheterization.

Our study found that the increase in age could increase susceptibility to becoming frail, which is consistent with the findings of previous studies. Casals and colleagues [16], Matsuoka and colleagues [17], and Tavares and colleagues [18] all reported a positive association between frailty and age, explaining that this association may be due to aging processes such as hormonal changes and lowered immunity. These processes expose elderly people to heart disease among multiple diseases, which decreases the ability of the heart to eject blood throughout the vascular system and hence leads to a decrease in blood supply to the body's systems.

As for the relationships between health variables and frailty, this study found that total cholesterol, triglycerides, RBS, and HDL were significantly and positively associated with frailty. Casals and colleagues [16] found that frail people had higher serum levels of total cholesterol, triglycerides, and HbA1c than did non-frail people. They explained that increased serum levels of total cholesterol, triglycerides, and HbA1c are risk factors for many vascular diseases that affect the body's main organs (i.e., heart, brain, and kidneys), which in turn predisposes people to frailty. Vascular diseases are characterized by the decrease of blood perfusion to the organs, resulting from either atherosclerosis or vasoconstriction [19].

This study found that participants with hypertension II (≥169/≥100 mmHg) had a higher tendency to be frail than did participants with normal blood pressure (100–129 to 60–89 mmHsg). In accordance with our study results, Liu and colleagues [20] and Vetrano and colleagues [21] found a positive relationship between frailty and hypertension. Meanwhile, Busby and colleagues [22] and Casals and colleagues [16] found a positive relationship between frailty and hypotension. We suggest that as people begin to be frail, they may experience increased peripheral vascular resistance (hypertension). Later, the myocardium muscle strength is decreased, and blood pressure is therefore decreased as well. What supports our assumption is that the participants in the studies of Busby and colleagues [22] and Casals and colleagues [16] were aged older than 60 years, leading the researchers to conclude that frail people experience hypotension (during late stages of frailty). However, in our study, the participants were aged between 24 and 90 years (mean = 49.9, SD = 11.4). Also, in the study of Vetrano and colleagues [21], the participants were aged between 18 and 86 years. This association between frailty and hypertension identified in the present study and in the study of Vetrano and colleagues [21] may be explained by the inclusion of young participants in our studies, as frailty may begin at a young age.

The present study findings indicated that frailty had significant positive associations with HC and WC among CAD patients undergoing cardiac catheterization. Similarly, Zaslavsky and colleagues [23] found that WC (>88 cm) followed by WHR (>0.8) were superior to all BMI categories in detecting frailty. Zaslavsky and colleagues explained that BMI cut-off points may be less able to predict future morbidity (loss in physiological, mental, and psychological ability) in older adults than in younger adults. In addition, older adults' body composition changes towards a decrease in lean tissue and height and an increase in fatty tissue. Monteil and colleagues [24] found that obesity (BMI ≥30 kg/m^2^, or WC > 88 cm) is positively associated with frailty. They justified their results by pointing out that in older adults, height is decreased and abdominal girth is increased, which could result from the weakening of muscle and increase in the fat composition of the older body.

### 4.1. Clinical Implications

This study addressed numerous significant traditional obesity parameters and health and biomarkers that might assist healthcare providers, including physicians and nurses, in detecting patients with CADs who are at higher risk for frailty while undergoing cardiac catheterization. This might provide a better scope of frailty and tailor the appropriate interventions to those risky patients on a timely manner. Similarly to this implication, a previous study indicated that future studies of frailty should address the significance of health-function indicators in detecting elderly patients who at higher risk for frailty, and in turn, planning effective healthcare [25].

### 4.2. Limitations

The results of the current study should be used cautiously due to numerous limitations. This study should be replicated with different populations, particularly with variant level of cardiovascular risk profile and metabolic syndrome, which might provide inconsistent findings pertaining to frailty. Moreover, in this study, the traditional cardiovascular risk factors and other risk factors contributed to frailty for 32% in the established model. Consequently, there are numerous factors that might negatively affect the health status of those risky patients, and which in turn, further investigations are needed to address these factors.

## 5. Conclusion

The predictors of frailty were total cholesterol, HC, triglycerides, age, random blood sugar, WC, and HDL. Participants educated to primary school level had a higher tendency to be frail than did illiterate participants. Further, hypertension II participants (≥169/≥100 mmHg) had a higher tendency to be frail than did participants with normal blood pressure (100–129/60–89 mmHg). The findings of this study could help healthcare providers, including nurses, to identify the factors that could lead to frailty among CAD patients undergoing cardiac catheterization.

## Figures and Tables

**Table 1:** Sociodemographic and health variables for the study participants (*N* = 220).

	N (%)	Mean (SD)	Median (min, max)
*Gender*			
Male	161 (73.2%)		
Female	59 (26.8%)		

Age (years)		49.9 (11.4)	49 (24,90)

*Marital status*			
Single	53 (24.1%)		
Married	137 (62.3%)		
Divorced	15 (6.8%)		
Widower	15 (6.8%)		

*Employment*			
Employed	108 (49.1%)		
Not employed	68 (30.9)		
Retired	44 (20%)		

*Educational level*			
Illiterate	10 (4.5%)		
Primary school education	40 (18.2%)		
High school education	72 (32.7%)		
Bachelor's degree	56 (25.5%)		
Master's degree	27 (12.3%)		
Doctoral degree	15 (6.8%)		

*Smoking (cigarette/day)*		26 (14)	25 (0,60)
Nonsmokers (0)	84 (38.2%)		
Light smokers (<10)	10 (4.5%)		
Moderate smokers (10–20)	72 (32.7%)		
Heavy smokers (>20)	54 (24.6%)		

*Blood pressure*			
Normal (100–129/60–89)	44 (20%)		
Hypertension I (130–159/90–99)	80 (36.4%)		
Hypertension II (≥160/≥100)	96 (43.6%)		

*Daily activity (steps/day)*		3016 (1107)	3030 (789, 6100)
Sedentary life style (<5000 steps/day)	201 (91.4%)		
Borderline (5000–10000 steps/day)	19 (8.6%)		

*Low density lipoprotein*		148 (18.8)	148 (102, 200)
Optimal <100 mg/dl	22 (10%)		
Borderline (100–129 mg/dl)	75 (24.1%)		
High ≥130 mg/dl	123 (55.9%)		

*High density lipoprotein*		45 (4.8)	44 (34, 60)
Borderline	141 (64.1%)		
High	79 (35.9%)		

*Triglycerides*		188 (34.3)	180 (112, 277)
Optimal <100 mg/dl	37 (16.8%)		
Normal 100–149 mg/dl	39 (17.7%)		
Borderline 150–199 mg/dl	87 (39.5%)		
High ≥200 mg/dl	57 (26%)		

*HS-CRP*		5 (1.4)	5 (2, 9)
Low risk	42 (19.1%)		
High risk	145 (65.9%)		

*HbA1c*		7.1 (0.86)	6.9 (5, 9)
Optimal	42 (19.1%)		
Borderline	76 (34.5%)		
Diabetic	102 (46.4%)		

*Anxiety (score of 1-21)*		11.8 (2)	12 (6, 16)
Normal	14 (6.4%)		
Borderline	46 (20.9%)		
Abnormal	160 (72.7%)		

*Depression (score of 1-21)*		10.7 (2.3)	11 (5, 15)
Normal	38 (17.3%)		
Borderline	82 (37.3%)		
Abnormal	100 (45.5%)		

*Frailty (score of 1-15)*		3.5 (1.7)	3 (0, 10)
Not frail (score <5)	160 (72.7%)		
Frail (score ≥5)	50 (27.3%)		

HS-CRP: High-sensitivity C-reactive protein; HbA1c: hemoglobin A1c.

**Table 2:** Bivariate analysis for the statistical differences (Pearson's Correlation) between obesity parameters and frailty.

Obesity parameters	Frailty (Pearson's correlation value)	*p* value
Weight	0.17	**0.01**
Waist circumference	0.17	**0.01**
Hip circumference	0.24	**0.00**
Waist height ratio	0.11	0.1
Waist hip ratio	−0.02	0.72
Body adiposity index	0.14−	**0.03**
Body Mass index	0.01	0.87
Body shape index	0.003	0.97

**Table 3:** Forward regression between obesity parameters and sociodemographic and health variables and frailty.

Model		*R * ^2^ for the model (*p* value)	Standardized coefficient (*β*)	*P*	CI 95% LL	Ul
1	Total cholesterol	0.09 (<0.001)	0.29	<0.001	0.30	0.802

2	Total cholesterol	0.157	0.32	<0.001	0.36	0.85
	HC (cm)	(<0.001)	0.27	<0.001	0.35	1.01

3	Total cholesterol	0.205	0.365	<0.001	0.442	0.93
	HC (cm)	(0.001)	0.31	<0.001	0.456	1.107
	Triglyceridesc(mg/dl)		0.226	0.001	−0.472	−0.126

4	Total cholesterol	243	0.369	<0.001	0.456	0.933
	HC (cm)	(0.002)	0.337	<0.001	0.528	1.17
	Triglyceridesc(mg/dl)		0.215	0.001	−0.454	−0.155
	Age (years)		0.198	0.002	0.011	0.05

5	Total cholesterol	0.282	0.344	<0.001	0.412	0.882
	HC (m)	(0.002)	0.355	<0.001	0.58	1.21
	Triglyceridesc(mg/dl)		0.197	0.002	−0.427	−0.09
	Age (years)		0.257	<0.001	0.02	0.06
	RBS (mg/dl)		0.208	0.001	−0.024	−0.006

6	Total cholesterol	0.304	0.324	<0.001	0.376	0.844
	HC (cm)	(0.015)	0.625	<0.001	0.949	2.2
	Triglyceridesc(mg/dl)		0.24	<0.001	−0.49	−0.149
	Age (years)		0.254	<0.001	0.02	0.059
	RBS (mg/dl)		0.221	0.001	−0.025	−0.007
	WC (cm)		0.308	0.015	−1.37	−0.151

7	Total cholesterol	0.324	0.288	<0.001	0.304	0.78
	HC (cm)	(0.02)	0.62	<0.001	0.947	2.19
	Triglyceridesc(mg/dl)		0.25	<0.001	−0.495	−0.158
	Age (years)		0.245	<0.001	0.019	0.057
	RBS (mg/dl)		0.214	0.001	−0.024	−0.006
	WC (cm)		0.329	0.009	−1.41	−0.207
	HDL		-0.146	0.02	−1.26	−0.111

The last model explained 32.4% of the variation of frailty score (*p*=0.02).

## Data Availability

The data used to support the findings of this study can be requested from the first author upon reasonable request.
